# National assessment of emergency staff level of practice in the management of forensic evidence

**DOI:** 10.1093/fsr/owad024

**Published:** 2023-08-11

**Authors:** Saad B Albishri, Fahed A Albednah, Nawaf S Alenazi, Nahaa E Alsubaie, Osama S Elserafy

**Affiliations:** College of Medicine, Imam Mohammad Ibn Saud Islamic University (IMSIU), Riyadh, Saudi Arabia; College of Medicine, Imam Mohammad Ibn Saud Islamic University (IMSIU), Riyadh, Saudi Arabia; College of Medicine, Imam Mohammad Ibn Saud Islamic University (IMSIU), Riyadh, Saudi Arabia; Department of Mathematics, AlKhurmah University College, Taif University, Taif, Saudi Arabia; Department of Forensic Medicine and Clinical Toxicology, Faculty of Medicine, Cairo University, Giza, Egypt; Department of Criminal Justice and Forensics, King Fahad Security College, Riyadh, Saudi Arabia

**Keywords:** forensic evidence collection, forensic education, forensic training, forensic nursing, forensic emergency medicine, guidelines

## Abstract

The emergency room is the most likely location where victims of violent crime would be encountered by the healthcare system, as the emergency staff is the first to evaluate the victim or culprit, exposing them to a range of forensic evidence. Forensic evidence can help exclude, identify, and prosecute a suspect and is classified as informational or physical evidence. Emergency staff must be proficient and knowledgeable in gathering, preserving, and documenting forensic evidence in their practice. To our knowledge, this is the first study that assesses the emergency staff’s level of practice in managing forensic evidence. The aims of this study are to assess the level of practice of emergency staff in managing forensic evidence and observe an association between emergency experience and the level of practice in managing forensic evidence, study a connection between forensic education/training and the level of practice in the management of forensic evidence. This observational cross-sectional analytical study in Saudi Arabia was conducted from January 2022 to December 2022. Participants completed a self-administered online survey. Measuring the level of practice was implemented through a researcher-designed questionnaire based on a paper that provided guidelines for forensic evidence collection in the emergency department. Most emergency healthcare workers had a good level of practice in managing forensic evidence (64.7%). Those with excellent practice scored the lowest in documentation, whereas participants in the poor practice category scored the lowest in the trace evidence and clothes domains. Emergency workers who encountered less number of forensic cases per month, i.e. less than two or three to five cases, were found to be more likely to have poor management of forensic evidence. Emergency personnel with no prior education or training are more likely to engage in poor practice in forensic evidence collection. Furthermore, those who had acquired forensic education/training had higher percentages of excellent forensic practice (56.52%) compared to poor practice (7.14%). Those who claimed that their institution had issued guidelines were more likely to have excellent practice (75.36%), whilst those who did not receive guidelines were more likely to have poor forensic evidence management (85.71%). More research is required involving local hospitals and utilizing consistently validated methods in evaluating forensic evidence collection.

**Key points:**

- A national assessment of emergency staff level of practice in the management of forensic evidence was performed.
- Most emergency staff had a good level of practice in the management of forensic evidence.
- More training and education are needed for emergency staff in the field of forensics.
- National evidence-based guidelines for managing forensic evidence in the emergency setting should be established.

## Introduction

Violent crime is rising worldwide and is now the fourth leading cause of death [1]. Many assault victims with physical injuries and those with various forms of physical, sexual, and psychological abuse require emergency room (ER) treatment. The primary goals of emergency medicine are to manage and treat patients. In contrast, the primary goals of forensic medicine are to medicolegally evaluate victims or culprits, recover evidence, write medicolegal reports, and, if necessary, testify in court [2]. However, the ER remains the most likely location where victims would be encountered by the healthcare system, as the emergency staff is the first to evaluate the victim or culprit, exposing them to a range of forensic evidence. Forensic evidence can help exclude, identify, and prosecute a suspect and is classified as informational or physical evidence. Informational evidence consists of clinical notes, injury documentation, body diagrams, photos, and observations. On the contrary, physical evidence is often tangible or measurable, and it can include trace evidence, which is minuscule or microscopic evidence that is not visible to the naked eye [3–5]. For evidence to be considered in court, the sequence of who has taken custody of it from the time it is collected until it is presented in court must be accurately documented, and this is referred to as the chain of custody [3,5]. If this chain is broken, the forensic evidence presented may very well be excluded from the legal case, which could negatively impact the victim. Therefore, emergency staff must be proficient and knowledgeable in gathering, preserving, and documenting forensic evidence in their practice. The utilization of forensic medical knowledge and proper requirements for living patients in the emergency department is termed forensic emergency medicine [6].

The level of competency of emergency physicians and other healthcare workers in dealing with medicolegal cases has been demonstrated in several international studies. The first study, conducted in the United States, included 134 emergency physicians and nurses from two urban level I trauma centres. This paper aimed to assess and compare emergency department nurses’ and physicians’ forensic practice, knowledge, and experiences. They discovered that just 13% of research participants had prior forensic training [7]. Moreover, a study was carried out amongst 175 nurses working in the emergency departments of 15 Turkish hospitals to determine how nurses approach forensics cases. The article revealed that none of the nurses had any forensics training, and 64.6% felt they lacked adequate knowledge in approaching and collecting forensics evidence [8]. Furthermore, a descriptive study of 44 healthcare workers in Bolu province, Turkey, attempted to assess emergency healthcare personnel’s knowledge and practices regarding protecting and preserving evidence in forensic cases. They observed that 90.9% of participants had managed forensic cases, 65.9% had no forensic training, and 22.7% were careless when removing and storing the patient’s garments [9].

Another study in Turkey examined 241 reports written by the Turkish Council of Forensic Medicine and compared them with reports written by emergency physicians for the same cases. The paper revealed that the address, time of examination, and time of the incident were not mentioned in more than half of the reports and concluded that physicians’ sensitivity and knowledge level regarding developing a forensic report was insufficient [10]. Likewise, a study was conducted amongst emergency nurses in New Zealand to know how closely they adhered to forensic standards and practices and the need for clinical forensic training for nurses working in the emergency setting. They discovered that both the chain of custody and the evidence collection were flawed. The requirement for all nurses working in the emergency department to complete postgraduate clinical forensic training programmes was received with explicit approval by the participants [11]. Globally, there are gaps in the literature regarding the level of practice of forensic evidence collection in the emergency department. Additionally, none of the studies used a consistently validated instrument to measure the management of medicolegal cases.

To our understanding, Saudi Arabia has yet to establish guidelines for managing forensic evidence in the ER. Additionally, no national study has been done to assess emergency staff’s level of practice in managing forensic evidence. However, few local papers have provided insight into the expertise of emergency staff in dealing with medicolegal matters. A study was conducted in four major hospitals in Jeddah, Saudi Arabia. The study’s goal was to assess emergency doctors’ awareness of legal and ethical features in managing medicolegal cases, assessment of physical and sexual abuse, photo-documentation, and management of forensic evidence. The article had 137 participants; although a large percentage of emergency physicians recognize the value of photography in documentation, many did not incorporate it into their practice (79.56%). Besides, almost half of the emergency physicians were confident that their workplace had a well-organized chain of custody for evidence collection (48.9%), and most of those who filled out the survey agreed that the current approach to medicolegal cases is flawed and needs to be improved through training and education [12].

Additionally, a study was performed in Dammam, Saudi Arabia, amongst 96 emergency nurses working in secondary hospitals to evaluate their knowledge and attitude towards forensics cases; the paper revealed that most nurses lacked basic forensic knowledge, and 69% of the nurses agreed that training in approaching forensic evidence was necessary [13]. Finally, a centre-based retrospective study was conducted at King Fahad Hospital in Dammam to assess the background of medicolegal injuries and identify any errors encountered by physicians in writing medicolegal reports; 418 medicolegal cases were included. Most cases were due to blunt injury 81.8%, with physical assault being the most common cause. The article noted that the time of admission was not written in all of the reports, and there was no mention of the size of the injury in almost all of the cases [14]. Overall, the findings support the notion that emergency healthcare workers in Saudi Arabia lack training and education concerning the management and documentation of medicolegal cases. The level of competency in the management of forensic evidence was not evaluated in any of the articles. Another limitation is that the papers used various methodologies and involved different populations, making comparisons between their results unfeasible. As a result, more research is needed in Saudi Arabia to evaluate emergency healthcare workers’ practices in dealing with forensic evidence.

This study has three aims. The first is to assess the level of practice of emergency staff in Saudi Arabia with regard to the management of forensic evidence. The second is to observe an association between emergency experience and the level of practice in managing forensic evidence. The third is to evaluate a connection between forensic education/training and the level of practice in the management of forensic evidence.

## Methods

### Study design

This observational cross-sectional analytical study in Saudi Arabia was conducted from January 2022 to December 2022. The study population consisted of emergency healthcare workers living in Saudi Arabia. A convenience sampling method was implemented. Data collectors were recruited to cover the five geographical regions of Saudi Arabia (Central, Eastern, Western, Northern, and Southern). Data collectors directly contacted ER staff through ER visits, social media, and messaging apps. Weekly meetings were held with the data collectors to monitor their progress and to discuss where the obtained data originated from, such as which hospitals were visited.

### Data collection tool

**Figure 1:**
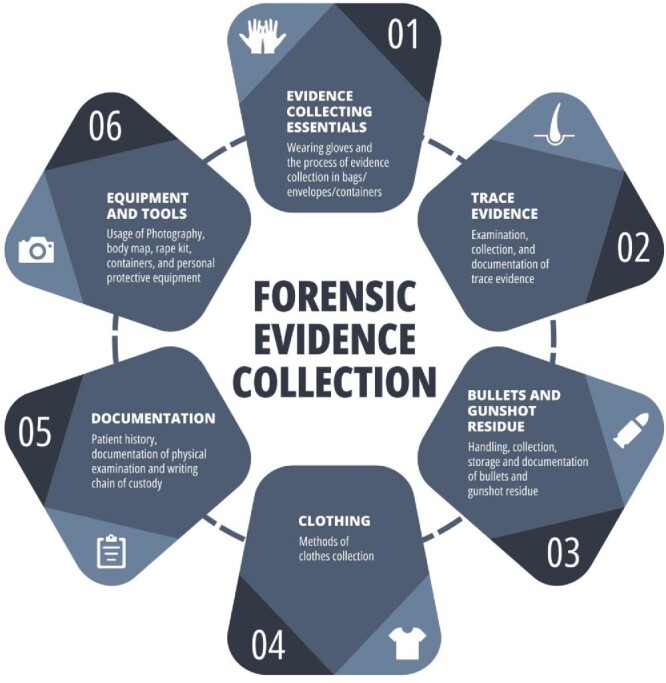
A brief description of the six forensic evidence collection domains that were utilized in the assessment.

Participants filled out an online self-administered survey. The survey was divided into three sections; the first section includes the participants’ personal data as well as information about their work experience. The second section contains information about their forensic education/training and experience. The third section assesses emergency healthcare workers’ level of practice in managing forensic evidence through a researcher-designed questionnaire. This part of the survey was structured based on guidelines for forensic evidence collection in the emergency department, published in 2010 [15]. The guidelines were developed using a multidisciplinary team, in which they gathered evidence from more than 20 articles in addition to consultations with law enforcement officials and forensic experts. The third section of the questionnaire was developed to include six domains: evidence-collecting essentials, trace evidence, bullets and gunshot residue, clothing, documentation, and equipment. Information regarding each domain can be seen in Figure 1. Each domain originally consisted of five questions, for a total of 30 questions. The Likert scale of frequency was used for each item. The answers included “always”, “often”, “sometimes”, “rarely”, and “never”, with the values assigned to each item ranging from 0 for “never” to 4 for “always”. Out of the 30 questions, items 5, 13, 16, 17, and 23 were used to highlight bad practices in handling forensic evidence, and in these questions, the scoring was reversed. Finally, each participant’s total score was assigned to one of four categories: “excellent practice”, “good practice”, “fair practice”, or “poor practice”.

### Pilot testing

#### Face validity

Face validity was implemented through a review of the survey questions by experts, who provided input on the comprehensiveness and readability of the questions. Based on feedback, changes to the grammar and the choice of specific vocabulary regarding forensic terminology were made.

#### Content validity

After establishing face validity, the questionnaire was referred to three different forensic medicine consultants, who were asked to read the objectives and thoroughly review the questionnaire by utilizing three measures “essentiality”, “relevance”, and “clarity”. The essentiality of each item was rated as either “essential”, “not essential but useful”, or “not essential”. The relevance and clarity of each item were rated on two separate scales as either “highly relevant/clear”, “quite relevant/clear”, “somewhat relevant/clear”, or “not relevant/clear”. Using the Lawshe technique, we measured the content validity of each item using the content validity ratio, item-level content validity index, and modified kappa statistics. The content validity process eliminated items 13, 16, and 17.

#### Reliability

A pilot study involving 55 emergency staff was carried out. Participants filled out the pre-final version of the survey. The internal consistency of the 30-item Likert-like scale (α = 0.77) was in the “acceptable” range [16]. Items 13, 16, and 17 were excluded from the questionnaire as was recommended in the content validity, which increased the Cronbach’s alpha score to α = 0.83.

### Ethical consideration

All writing is done in accordance with the fundamental ethical standards and policies of the Institutional Review Board (IRB) at Imam Muhammad Ibn Saud Islamic University, College of Medicine, following the ethical principles of the Declaration of Helsinki. The project was approved by the IRB at Imam Muhammad Ibn Saud Islamic University, College of Medicine, No. 321/2022. The survey link was attached with a brief overview of the study and its purpose, with a more detailed explanation on the survey’s web page. Participants were told that filling out the survey constituted consent to be enrolled in the study.

### Statistical analysis

We used IBM SPSS version 28 to conduct descriptive statistical analysis and chi-square tests of independence. Findings are reported in weighted percentages, and the reported number is adjusted using the weighted analysis. The chi-square test of independence was used to evaluate the association of forensic practice groups and other variables. Missing values were handled *via* case-wise deletion, and *P*-values <0.05 were considered statistically significant.

## Results

Four hundred and fifty participants completed the survey. Respondents were comprised of 54.7% males and 45.3% females, with the majority being Saudi (86.4%) and residing in the Eastern region of Saudi Arabia (39.1%). Most of the participants (58.0%) held a Bachelor’s degree. Concerning respondents’ occupations, 240 (53.3%) were physicians, and 179 (39.8%) were nurses. A large number of our samples were employed in the Ministry of Health hospitals (186, 41.3%). A comprehensive presentation of the demographic characteristics can be found in Table 1.

**Table 1:** Descriptive analysis of the participants sociodemographic characteristics (*N* = 450).

Variable	*n* (%)
Sex	
Male	246 (45.3)
Female	204 (54.7)
Nationality	
Saudi	389 (86.4)
Non-Saudi	61 (13.6)
Residence	
Central region	83 (18.4)
Eastern region	176 (39.1)
Northern region	82 (18.2)
Southern region	74 (16.4)
Western region	35 (7.8)
Education	
Diploma	41 (9.1)
Bachelor’s degree	261 (58.0)
Master’s degree	67 (14.9)
Doctorate degree	81 (18.0)
Physician	240 (53.3)
Occupation	
Nurse	179 (39.8)
Paramedic	25 (5.6)
Others	6 (1.3)
Ministry of Health hospitals	186 (41.3)
Hospital type	
University Hospital	96 (21.3)
National guard hospitals	59 (13.1)
Armed forces hospitals	28 (6.2)
Security forces hospitals	27 (6.0)
Private hospitals	54 (12.0)
Professional level	
Physician professional level	
Intern	12 (2.7)
Resident	78 (17.3)
Specialist	38 (8.4)
Fellow	78 (17.3)
Consultant	34 (7.6)
Non-physician professional level	
Specialist	118 (26.2)
Specialist first class	40 (8.9)
Specialist consultant	52 (11.6)

Percentage may not sum up to 100% due to rounding.

Most of our sample had an experience in the ER of either <1 year (32.7%) or 5–9 years (31.8%). Two hundred thirty-three (51.8%) had previous forensic education/training, with the majority having it at the undergraduate level (131, 29.1%), (46, 10.2%) at the postgraduate level, and 56 (12.4%) at both levels. It was reported that 184 (40.9%) of those with prior forensic education/training had specialized education/training in the management of forensic evidence in ER settings. Most physicians in our sample received prior forensic education/training (139/240, 57.92%), compared to only 44.69% (80/179) of nurses. Two hundred sixty-three (58.4%) reported that their hospital had guidelines for dealing with forensic evidence. The participants’ experiences and forensic education/training are outlined in Table 2.

**Table 2:** Descriptive analysis of the participants’ experience and forensic education/training (*N* = 450).

Variable	*n* (%)
Experience in emergency (ER)	
< 1 year	147 (32.7)
1–4 years	96 (21.3)
5–9 years	143 (31.8)
10–14 years	33 (7.3)
≥ 15 years	31 (6.9)
Previous forensic training/education	
No	217 (48.2)
Yes	233 (51.8)
Forensic training/education level	
Both	56 (12.4)
Post-graduate	46 (10.2)
Undergraduate	131 (29.1)
Forensic training/education in forensic evidence collection in the ER setting	
No	49 (10.9)
Yes	184 (40.9)
Forensic cases managed per month	
≤ 2	241 (53.6)
3–5	133 (29.6)
6–8	57 (12.7)
≥ 9	19 (4.2)
Guidelines	
No	187 (41.6)
Yes	263 (58.4)

Percentage may not sum up to 100% due to rounding.

Most participants fell into the “good practice” category (291/450, 64.7%). The level of forensic practice was analyzed through the bivariate analysis for statistical associations with the other demographics, training, and experience variables. A complete description of the associations is presented in Table 3. There is a significant association between forensic practice and experience in ER (*P* = 0.003). A significant link between forensic evidence handling and prior forensic education/training can be seen (*P* = 0.008). According to the chi-square test of association, those with no prior education/training are more inclined to have poor practice (92.86%). In addition, those who received previous forensic education/training were found to have more excellent forensic practice (56.52%) compared to poor practice (7.14%). The level of practice in handling forensic evidence and the presence of guidelines regarding managing forensic cases showed a statistically significant association (*P* < 0.001). Those who described their institution as providing guidelines were more likely to have excellent practice (75.36%), whilst those who did not have placed guidelines were more likely to be poor in managing forensic evidence (85.71%).

**Table 3:** Descriptive bivariate analysis of the participants’ level of practice with their sociodemographic and professional/academic characteristics (*N* = 450), *n* (%).

Variable	Excellent (*n* = 69)	Good (*n* = 291)	Fair (*n* = 76)	Poor (*n* = 14)	χ^2^ (*P-*value)
Sex					
Female (*n* = 204)	33(47.83)	128(43.99)	40(52.60)	3(21.43)	5.25(0.155)
Male (*n* = 246)	36(52.17)	163(56.01)	36(47.40)	11(78.57)	
Hospital type					
Armed forces hospitals (*n* = 28)	2(2.90)	23(7.90)	3(3.95)	0(0)	14.29(0.504)
Ministry of Health (*n* = 186)	36(52.17)	119(40.89)	27(35.53)	4(28.57)	
National guard (*n* = 59)	8(11.59)	38(13.06)	11(14.47)	2(14.29)	
Private hospitals (*n* = 54)	7(10.14)	31(10.65)	12(15.79)	4(28.57)	
Security forces hospitals (*n* = 27)	3(4.35)	19(6.53)	5(6.58)	0(0)	
University hospital (*n* = 96)	13(18.84)	61(20.96)	18(23.68)	4(28.57)	
Occupation					
Physician (*n* = 240)	36(52.17)	157(53.95)	37(48.68)	10(71.43)	9.82(0.365)
Nurse (*n* = 179)	29(42.03)	115(39.52)	31(40.79)	4(28.57)	
Paramedic (*n* = 25)	4(5.80)	13(4.47)	8(10.53)	0(0)	
Others (*n* = 6)	0(0)	6(2.06)	0(0)	0(0)	
Experience in the ER					
< 1 year (*n* = 147)	33(47.83)	82(28.18)	28(36.84)	4(28.57)	30.06(**0.003**)
1–4 years (*n* = 96)	10(14.49)	71(24.40)	7(9.21)	8(57.14)	
5–9 years (*n* = 143)	3(4.35)	21(7.22)	7(9.21)	0(0)	
10–14 years (*n* = 33)	20(28.99)	94(32.30)	27(35.53)	2(14.29)	
≥ 15 (*n* = 31)	3(4.35)	23(7.90)	7(9.21)	0(0)	
Previous Forensic Training/Education					
No (*n* = 217)	30(43.48)	138(47.42)	36(47.37)	13(92.86)	11.89(**0.008**)
Yes (*n* = 233)	39(56.52)	153(52.58)	40(52.63)	1(7.14)	
Forensic Training/Education level					
Both (*n* = 56)	10(25.64)	34(22.22)	11(27.50)	1(100.00)	5.09(0.533)
Post-graduate (*n* = 46)	8(20.51)	33(21.57)	5(12.50)	0(0)	
Undergraduate (*n* = 131)	21(53.85)	86(56.21)	24(60.00)	0(0)	
Forensic Training/Education in forensic evidence collection in the ER setting					
No (*n* = 49)	11(28.21)	25(16.34)	12(30.00)	1(100.00)	8.93(**0.030**)
Yes (*n* = 184)	28(71.79)	128(83.66)	28(70.00)	0(0)	
Guidelines					
No (*n* = 187)	17(24.64)	121(41.58)	37(48.68)	12(85.71)	20.96(**<0.001**)
Yes (*n* = 263)	52(75.36)	170(58.42)	39(51.32)	2(14.29)	
Forensic cases managed per month					
≤ 2 (*n* = 241)	45(65.22)	151(51.89)	33(43.42)	12(85.71)	21.64(0.010)
3 to 5 (*n* = 135)	15(21.74)	96(32.99)	20(26.32)	2(14.29)	
6 to 8 (*n* = 57)	7(10.14)	33(11.34)	17(22.37)	0(0)	
≥ 9 (*n* = 19)	2(2.90)	11(3.78)	6(7.89)	0(0)	

Prior education/training in forensic evidence collection in the emergency setting converged significantly with excellent practice (71.79%) compared to poor practice (0%) (*P* = 0.030). Professionals who encountered less number of forensic cases per month, i.e. less than two and three to five cases, were found to be more predicted to have poor handling of forensic evidence. An average score of each domain showed that those with excellent practice scored the lowest in documentation, whereas participants in the poor practice category scored the lowest in the trace evidence and clothes domains (data not shown).

## Discussion

This study aimed to establish the level of practice in handling forensic evidence by emergency staff in Saudi Arabia and assess a connection between the level of managing forensic evidence collection with experience in the ER and forensic education/training. The majority had “good practice” (64.7%). A significant link was observed between both ER experience and forensic education/training with the quality of handling forensic evidence.

Results comparability with existing international papers was challenging due to the utilization of different methodologies in each article to measure different outcomes. Moreover, each nation has its unique medicolegal procedures and administration, which might make it difficult to compare and contrast findings. Similar to previous research, we recognized no significant link between sex and forensic practice in our investigation [7,17]. In addition, 51.8% of the total sample had prior forensic education/training. When divided by occupation, we observe that physicians had 57.92% and nurses had 44.69% previous training/education. These results are greater than what is currently reported in the literature [7–9]. In a published article, forensic knowledge was evaluated, and the average scores for nurses and physicians, out of a maximum of 50 points, were 37.7 and 40.1, respectively [7]. Despite the fact that this cannot be compared to our scoring and category of forensic practice, it can be associated as a factor impacting the practice, as their knowledge level appears to be adequate. More studies should be implemented to determine the association between knowledge, attitude, and practice level. Documentation errors proved to be a vital issue in the literature [10,18]. This observation coincided with our result, as even those who showed excellent results scored the lowest average in the documentation domain. To our knowledge, this is the first article to assess emergency staff’s handling of forensic evidence through an extensive review of their practices.

Saudi Arabia is a growing nation with a population of around 35 million [19]. With this growth comes an increase in violent crime [20]. Many studies have been conducted throughout the nation exploring topics involving homicide, suicide, stab injuries, firearm fatalities, and domestic and child abuse [21–29]. Such cases are more likely to end up in the ER, and it is up to the emergency staff to manage them. Our study findings were based on classifying specific aspects of forensic evidence collection into domains that provide a broader picture of current practice. On the other hand, the local survey of Jeddah hospitals yielded results that covered only a few particular topics, such as photography. Moreover, when comparing the level of documentation, more than half of the participants (68.6%) claim to provide adequate documentation [12]. Yet, in our study, individuals with excellent practice performed the worst in the documentation domain. This disparity in findings could be due to Zaki’s study asking participants if they thought their documentation was adequate, which does not highlight the real-life practice. Overall, the study did not explore other aspects of forensic evidence collection, making comparisons with our findings inaccurate; therefore, more local or institutional studies should be implemented to evaluate forensic evidence collection in emergency settings.

In Riyadh, Saudi Arabia, a descriptive study comprising different healthcare centres was done to assess the medical staff’s medicolegal knowledge, attitude, and practice (KAP) to identify the impact of experience, education, and training on medical practitioners’ medicolegal KAP. According to the survey, there were statistically significant variations between years of experience and individual factors, as well as the cumulative level of knowledge, attitude, and practices [30]. Likewise, our findings revealed a statistically significant relationship between emergency years of experience and level of forensic evidence management. However, comparing and contrasting the conclusions of both studies is problematic since the Riyadh study evaluates the KAP and correlates it with years of practice, but our study exclusively measures and correlates the level of practice with years of experience.

### Education and training

According to our findings, 233 participants had prior education/training in managing forensic evidence, with the bulk of their education being at the undergraduate level and 79% (184/233) having training in managing forensic evidence in emergency settings. Similarly, 87% of Zaki’s study participants had previous forensic education at the undergraduate level. However, the overwhelming majority of their respondents received no forensic training and were never involved in any evidence collection training programme in the emergency department [12]. This discrepancy in findings could be because the study only included a few local hospitals in the city of Jeddah compared to our national survey. In our study, participants with no prior forensic education/training are more likely to engage in poor practice of forensic evidence collection (*P* = 0.008). Furthermore, those with previous forensic education/training were shown to have more excellent practice compared to poor practice managing forensic evidence (*P* = 0.008). Prior education/training in forensic evidence collection in the emergency setting correlates significantly with having excellent practice compared to having poor practice in forensic evidence collection (*P* = 0.030). These findings highlight the importance of education and training of emergency healthcare workers. In Saudi Arabia, only 16 universities provide undergraduate courses in forensic medicine, with all of them teaching it to medical students exclusively [31]. To our understanding, no postgraduate programmes exist in Saudi Arabia besides the Saudi board residency training programme for forensic medicine. This means that for the rest of the healthcare occupations, educational courses, or programmes incorporating the study of forensics are not offered. Therefore, more undergraduate and postgraduate academic programmes should be implemented for various medical specialties. Any person contributing to the gathering and transferring of evidence could be asked to submit a report or provide testimony in court. Unfortunately, when providing patient care, an emergency physician who lacks forensic training could unintentionally overlook, misplace, or destroy crucial gross and trace evidence. Although they may be expected to provide “expert” forensic opinions, emergency physicians typically have little forensic training [32]. There is a gap in the curriculum for emergency medicine residents regarding clinical forensic medicine. Determining the best teaching methodology and structure for emergency medicine residents will require further research. The consensus model of emergency medicine clinical practice developed by major emergency medicine organizations must include the fundamentals of clinical forensic medicine [32]. Currently, in Saudi Arabia, mandatory courses or workshops are not incorporated in emergency residency programmes for managing forensic evidence; thus, efforts to mitigate these challenges must be more effective. It is vital that emergency physicians obtain training on how to undertake a clinical forensic evaluation, including how to execute an adequate forensic examination, evidence collecting, documentation, and the duty of the emergency physician in managing forensic cases.

### Guidelines

According to our survey results, 58.4% of participants reported having forensic evidence collection guidelines. In the Jeddah report, 68.6% of respondents said their workplace had a specific protocol for collecting evidence from forensic cases [12]. According to our findings, emergency personnel who described their institution as offering guidelines were more likely to have good practice, whereas those who did not have guidelines were more likely to have poor practice in managing forensic evidence (*P* < 0.001). This emphasizes the significance of guidelines in the emergency department for managing forensic cases, specifically in collecting forensic evidence. Based on this paper and the existing literature, institutional guidelines exist in most emergency departments. However, inconsistency between the guidelines may result in certain aspects being neglected. Hence, consensus guidelines should be devised to avoid errors or discrepancies. There should be a combined multidisciplinary effort from medical emergency experts and forensic experts, with consultations of law enforcement officials to establish national evidence-based guidelines for forensic evidence collection in hospitals.

### Forensic nursing

Forensic nursing was first acknowledged in the USA in 1990 [33]. The forensic nurse works as a link between doctors and the criminal justice system. As the patient’s initial point of contact, the forensic nurse is responsible for adequately assessing and gathering evidence that could jeopardize the legal process. A forensic nurse’s other responsibilities include preserving the chain of custody, treating trauma patients, and suspecting violence [34]. The study in Dammam showed that only 34% of nurses were aware of the term “forensic nursing”, with half of the nurses willing to practice forensic nursing if it existed [13]. Therefore, it is crucial to develop a programme for nurses in the specialty of forensic nursing and increase awareness of their role. The duty of emergency nurses in approaching forensics cases is crucial as most of the victims of violence and crime are first assessed and treated by ER nurses. Nurses working in the ER must have excellent forensic knowledge, particularly regarding the preservation and collection of evidence, in addition to their primary responsibilities in the life-saving management and resuscitation of patients. Accordingly, all nurses working in ER should be able to provide the best medical and legal care by identifying, collecting, documenting, and preserving evidence that can be presented to law enforcement authorities [35]. Many studies in different countries have implied the lack of forensics knowledge amongst nurses in the ER [36–39]. In Alsaif’s paper, nearly all emergency nurses received no lectures or training in dealing with forensic cases. A total of 77% agreed that they are not adequately trained in the field of forensics [13]. Our article has found that only 44.69% of nurses have received prior forensic education/training, and most emergency staff with excellent practice in managing forensic evidence have had forensic education/training in the ER. For this reason, it is necessary to create a mandatory educational training course for emergency nurses that reflects Saudi Arabia’s prevalence of forensic cases.

### Recommendations

Based on the findings of our assessment and the current state of the literature, we recommend to:

- Develop consensus nationwide evidence-based guidelines for managing forensic cases in the emergency setting.
- Establish an official conjoint forensic nursing programme in Saudi Arabia.
- Initiate mandatory forensic rotations or courses as part of the emergency residency programme for physicians.
- Organize and introduce continuous medical education of forensic courses, lectures, and workshops for emergency healthcare workers.
- Incorporate forensic medicine curriculum in undergraduate degrees of nursing, paramedics, etc*.*
- Modify undergraduate forensic medicine curriculums to be more healthcare and hospital oriented.
- Expand the number of forensic medicine departments and personnel available to provide forensic education in undergraduate healthcare specialties.

### Limitations

This paper has some limitations that should be emphasized. There is a possibility of self-report or recall bias because the participants provided the information. Since most emergency healthcare workers do not consider it part of their responsibility, getting an exceptionally high response rate was challenging. Additionally, the forensic role of emergency healthcare workers is somewhat novel and controversial, and their involvement in forensic cases is sometimes viewed with resentment since it frequently requires legal work that takes unpaid time away from practice and residency training [32]. Moreover, comparison with other research was not possible because this is the first study that uses a validated method across the country. The lack of universal guidelines makes it difficult to accurately assess the real-life practice of forensic evidence collection in the emergency setting.

## Conclusion

The majority of emergency workers had a good level of practice when dealing with forensic evidence. Emergency personnel with no prior education or training are more likely to engage in poor practice in forensic evidence collection. Furthermore, those who had acquired forensic education/training had higher percentages of excellent forensic practice compared to poor practice. Those who claimed that their institution had issued guidelines were more likely to have excellent practice, whilst those who did not receive guidelines were more likely to have poor forensic evidence management. All in all, more research is required involving local hospitals and utilizing consistently validated methods in evaluating forensic evidence collection.
